# Supplementary material for: Yearling proportion correlates with habitat structure in a boreal forest landbird community
Source: PeerJ. 2020 Apr 24;8:e8898. doi: 10.7717/peerj.8898 (PMC7185041; doi:10.7717/peerj.8898)
Supplement: Supplemental Information 1 [file peerj-08-8898-s001.pdf]

Supplementary Information for:

Correlating landbird yearling proportions with habitat quality and reclamation maturity using a multi-species approach

Peter Pyle[1,*], Kenneth R. Foster[2], Christine M. Godwin[2], Danielle R. Kaschube[1], and James F. Saracco[1]

[1]The Institute for Bird Populations, Point Reyes Station, 94956, USA
[2]Owl Moon Environmental Inc., Fort McMurray, T9K 0R3, Canada
*ppyle@birdpop.org

Analysis details for multi-species hierarchical model of yearling probability responding to vegetation greenness. We implemented the hierarchical model using Bayesian methods in JAGS[1] from R[2] using the R package "'jagsUI"[3]. We assigned vague normal priors of Norm(0, 1000) to mean hyperparameters and U(0, 10) for standard deviations. We obtained posterior distributions by sampling full conditional distributions using MCMC methods[4]. Posteriors were based on 30,000 iterations of three chains after an adaptive phase of 40,000 iterations and then discarding the next 10,000 iterations and thinning by three. Models were considered to have converged based on values for all parameters < 1.1[5]. We assessed the model with a $\chi2$ goodness-of-fit test and Bayesian p-value that compared model fit to the data to fit from data sets generated from predicted values. This test suggested adequate fit (Bayesian p = 0.492).

JAGS code for the model and goodness-of-fit test are given below.

Literature Cited

1. Plummer, M. JAGS: A program for analysis of Bayesian graphical models using Gibbs sampling. *Proc. 3rd Int. Workshop on Distrib. Statist. Comput.* (Vienna, Austria, 2003).

2. R Core Team. R: A language and environment for statistical computing (R Foundation for Statistical Computing, 2015).

3. Kellner, K. jagsUI: A Wrapper Around "rjags" to Streamline "JAGS" Analyses. R package 1.3.7. http://CRAN.R-project.org/package=jagsUI (2015).

4. Gilks, W. R., Richardson, S., & Spiegelhalter, D. J. In Markov Chain Monte Carlo methods in practice (Chapman and Hall, 1996).

5. Gelman, A. & Hill, J. Data analysis using regression and multilevel/hierarchical models (Cambridge University Press, 2006).

JAGS code for the model and goodness-of-fit test

```
 model{
   # priors
   for (i in 1:nspec){
    a0[i] ~ dnorm(mu.spec, tau.spec) # random species intercepts
    a.evimn[i] ~ dnorm(mu.evimn, tau.evimn)
    }
    mu.spec ~ dnorm(0, 0.001)
    tau.spec <- 1/(sigma.spec*sigma.spec)
    sigma.spec ~ dunif(0,10)
    mu.evimn ~ dnorm(0,0.001)
    tau.evimn <- 1/(sigma.evimn*sigma.evimn)
    sigma.evimn ~ dunif(0,10)

    # likelihood
    for (i in 1:nind){
    sy[i] ~ dbern(psy[i])
    logit(psy[i]) <- a0[spec[i]] + a.evimn[spec[i]]*evimn[i]

    # goodness-of-fit
    predicted[i] <- psy[i]
    res.sy[i] <- ((sy[i] - predicted[i]) / sqrt(predicted[i]*(1-predicted[i])))^2

    sy.rep[i] ~ dbern(psy[i])
    res.sy.rep[i] <- ((sy.rep[i] - predicted[i]) / sqrt(predicted[i]*(1-predicted[i])))^2
    }
   fit <- sum(res.sy[])          # test statistic for data
```

```
fit.rep <- sum(res.sy.rep[])   # test statistic for new predicted data

test <- step(fit.rep - fit)   # Test whether new data set more extreme

bpvalue <- mean(test)     # Bayesian p-value

}
```
